# Indications of digital literacy during Latino-focused, community-based COVID-19 testing implementation

**DOI:** 10.1093/jamiaopen/ooae115

**Published:** 2024-11-11

**Authors:** Patric V Prado, Carina Arechiga, Kara Marson, Yolanda Oviedo, Tatiana Vizcaíno, Monica Gomez, Arandu Alvarez, Laura Jimenez-Diecks, Sindy Guevara, Alexandra Nava, Zully Lopez, Omar Carrera, Robert Hypes, Carina Marquez, Gabriel Chamie

**Affiliations:** Department of Medicine, University of California, San Francisco, San Francisco, CA 94110, United States; Department of Medicine, University of California, San Francisco, San Francisco, CA 94110, United States; Department of Medicine, University of California, San Francisco, San Francisco, CA 94110, United States; Canal Alliance, San Rafael, CA 94901, United States; United Way of Merced, Merced, CA 95340, United States; United Way of Merced, Merced, CA 95340, United States; Canal Alliance, San Rafael, CA 94901, United States; Canal Alliance, San Rafael, CA 94901, United States; Canal Alliance, San Rafael, CA 94901, United States; Canal Alliance, San Rafael, CA 94901, United States; Canal Alliance, San Rafael, CA 94901, United States; Canal Alliance, San Rafael, CA 94901, United States; United Way of Merced, Merced, CA 95340, United States; Department of Medicine, University of California, San Francisco, San Francisco, CA 94110, United States; Department of Medicine, University of California, San Francisco, San Francisco, CA 94110, United States

**Keywords:** informatics, digital literacy, community health worker, COVID-19

## Abstract

**Objective:**

We sought to characterize indicators of digital literacy among persons testing for COVID-19 and community health workers (CHWs) providing testing via a digital platform in low-income, majority-Latino communities in California.

**Materials and Methods:**

From March 2021 to March 2022, we trained CHWs to provide community-based COVID-19 testing that relied on a digital platform for registration, recording and reporting of results. Among community members, we examined factors associated with accessing test results digitally and time to results receipt. Among CHWs, we evaluated factors associated with self-reported difficulty using the digital platform and improvement post-training.

**Results:**

Overall, 5044 community members were tested for COVID-19. Accessing results digitally vs non-digitally was associated with younger age (Odds Ratio [OR]: 1.02 [95% Confidence Interval [CI], 1.01-1.03], for each year decrease), rural residence (OR:1.61 [95% CI, 1.30-1.99]), and providing an email address at registration (OR: 2.18 [95% CI, 1.80-2.65]). The likelihood of providing an email address at registration was increased among younger, non-Latino, English-speaking, female, and rural testers. Among persons accessing results digitally, median time from testing to result receipt was 41 min, with increased time associated with rural residence and older age. Among 42 CHWs surveyed, 29 (68%) reported technology-related challenges when providing testing: those reporting challenges were more likely to be older and rural CHWs. Rural CHWs were less likely to report technical skill improvement post-training.

**Discussion:**

Email provision may be an indicator of digital literacy among persons testing for COVID-19 in low-income, majority-Latino communities. Rural and older CHWs may need more intensive digital training.

**Conclusion:**

Efforts to improve digital literacy in underserved communities are likely needed to realize the full potential of community-based health interventions that utilize digital platforms.

## Introduction

Digital health technologies have played an important role in increasing the availability of COVID-19 testing services outside of medical settings in the United States. Supported by online platforms, these health technologies have included services such as test scheduling and real-time access to electronic test results, as well as reporting to departments of public health.^1^^,^^2^ National public health organizations have prioritized new paradigms in digital health (Public Health 3.0) to support health providers to digitize health data, enhance electronic health record infrastructure, and improve digital competencies with the goal of supporting healthy, equitable communities.^3^ Since early in the response to the COVID-19 pandemic, digital modes of COVID-19 testing service delivery, including scheduling and results disclosure, have allowed health systems to cope with COVID-19 testing surges, while also increasing result notification timeliness and completeness.^4^

However, the COVID-19 pandemic has also highlighted pre-existing health inequities, particularly among Latino and Black/African American communities in the United States.^5^ These inequities also extend to digital healthcare access (eg, availability of computers/phones and internet) and digital literacy (eg, ability to use technology), especially among older people and people of color.^6^^,^^7^ Unless addressed, these disparities in digital access and literacy could result in decreased access to healthcare and worsen health outcomes.^8^ For example, digital health access barriers may decrease access to antiviral medications, which have time-sensitive windows for administration and in turn may widen COVID-19 related health disparities, such as death and hospitalization outcomes.^9^ As health systems increasingly rely on digital technology to extend services outside of clinics (COVID-19 testing, accessing COVID-19 treatment via telehealth, etc), there is an urgent need to understand digital literacy and demographic-related disparities among persons seeking COVID-19 testing services.^10^

With community-based organizations (CBOs) and community health workers (CHWs) responding to localized community COVID-19 needs, including testing services, it is also important to understand digital literacy and barriers to digital technology use among CBO members and CHWs.^11^^,^^12^ While there is literature on interventions to bridge the digital divide in the era of COVID-19 for general populations^13–15^ and the public health workforce,^16^ there is a dearth of literature on digital literacy and digital training efforts to support the use of digital COVID-19 testing platforms by Latino, Spanish-speaking CHWs.

*Aliados por la Salud* is an academic-community partnership formed in January 2021 between researchers and Latino-focused CBOs in Northern California. The goal of the partnership is to promote cross-community collaboration to increase ease and accessibility of COVID-19 testing in 2 predominantly Latino districts: the Canal District, Marin County (89% Latino) and Planada, Merced County (98% Latino).^17^ A digital, web-based platform (PrimaryBio) has been central to COVID-19 testing implementation, including testing registration, sample and result tracking, results reporting to participants and local public health departments, and data management, using laptops and tablets. In the present study, we sought to define indicators of digital literacy in populations testing for COVID-19 and CBOs/CHWs implementing testing, evaluate demographic factors associated with these indicators, and assess potential public health impacts of observed associations. For those testing, we focused our analysis on factors related to the ability to receive digital COVID-19 results, whereas for CHWs we focused on the training and skills needed to deliver digitally-based COVID-19 testing and results.

## Methods

### The *Aliados por la Salud* testing program

We (*Aliados por la Salud*) assessed technology literacy and challenges with digital access of COVID-19 testing, from March 2021 to March 2022, during community-based COVID-19 testing campaigns in 2 predominantly Latino Communities. We offered low-barrier, community-based COVID-19 testing in the Canal District in Marin County starting in March 2021, and in Planada, Merced County starting in April 2021, with testing ongoing at present.^17^ Testing was initiated through mass testing campaigns aimed at accelerating community-wide education and engagement. This was extended to weekly “drop-in” testing at predesignated hours and days near CBO offices and at several locations throughout the community. To decrease testing barriers even further, appointment-based testing was added, along with targeted community business testing (for employees). The PrimaryBio digital, web-based platform was used for testing participant registration, sample and result tracking and quality control, results reporting to participants and local public health departments, and data/participant management, using laptops and tablets. Community members were required to provide a phone number, at minimum, so staff could reach them in the event of a positive result; providing an email was optional. Community members could also physically retrieve printed results, and/or provide landline phone numbers (ie, access results non-digitally), if they had any difficulty with digital access. To assist with testing, our community CBO partners identified and recruited CHWs.

### CHW digital training

When extending testing to CBO offices and community locations, we provided digital and technical training to CHWs who delivered testing for COVID-19 (for 1 full day before testing implementation), to ensure CHWs could assist with digital survey data entry (of persons testing for COVID-19) through tablets and with digital photographs of rapid test kit results (for quality control purposes), use label printers and barcode scanners to track tests and results, use laptops for registration and to record results, and use printers for providing paper-based COVID-19 results. These skills were routinely reinforced during subsequent retraining (typically 2-3 h, twice a year) and trainer-observed testing. Informal assessment of skills occurred during training, and we implemented frequent quality assurance monitoring of data to understand if correct procedures were being followed.

### Analysis for COVID-19 testing population

Our primary indicator of digital literacy (analyzed as a dependent variable) among the testing population was the proportion of persons testing for COVID-19 that provided an email address for accessing results, and our primary indicators of digital health technology use behaviors (analyzed as dependent variables) were the proportion who opened and viewed their results digitally (by Short Message Service (SMS) or email, as opposed to land-line phone calls or in-person at CBO offices), and time from testing to result receipt. Participants who did not access their results digitally were excluded from the analysis on time to receipt of results, due to lack of measurement of results receipt time with non-digital methods. We defined email address provision as a probable indicator of digital literacy, as it may identify individuals without access to email, given that email is a key means of digital communication and often required for engaging with websites/digital media.^18^ Similarly, we considered receipt of a digital health result as a probable digital literacy indicator, and more specifically a digital health technology use behavior, consistent with a recent systematic review that defined digital health literacy as, “the ability to find and use health information with the goal of addressing or solving a health problem using technology.”^19^ However, we acknowledge that health literacy is multi-faceted and incorporates many societal, behavioral, and educational factors that impact the trust, ability, and follow-through necessary to use technology in receiving these digital results. Planada was classified as rural and Canal as peri-urban. Some of the testing populations were volunteers and staff at the event, which was also included as an explanatory factor in analysis (CHW Affiliation). Because some participants may have tested multiple times over the year, we de-duplicated persons tested and only analyzed each participant’s first-time testing. Our digital testing platform (PrimaryBio) recorded the time from testing to most recent result receipt, meaning observations may have been skewed if participants decided to review their results again days or weeks after initial review. Because of this, we used quantile regression, which is more robust to outliers,^20^ to obtain adjusted median values.

### Analysis of digital training of CHWs

**Figure 1.**
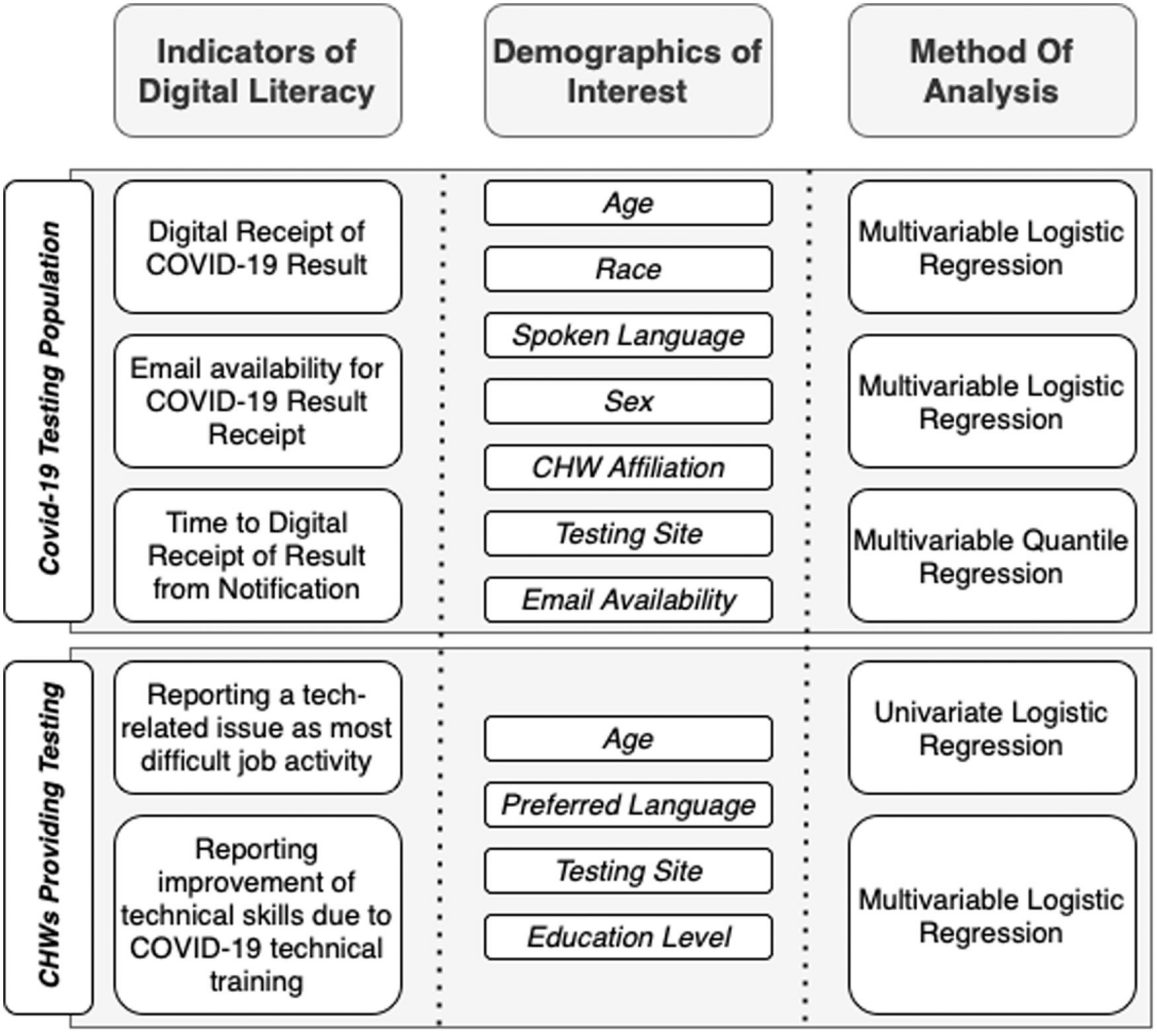
Study design with identified indicators (dependent variables), demographics of interest (independent variables), and methods of analysis.

We administered a survey in May 2022 to assess CHWs’ perceived difficulty in using technology and the impact of the training and testing implementation on their technical skills. All CHWs who were actively participating in testing at the time of survey implementation were eligible. CHW-reported challenges in implementing testing (dependent variable) were classified into digital technology-related (eg, registering participants into the PrimaryBio system after the survey, accessing/using surveys, using tablets/laptops) and non-digital technology-related challenges (eg, using/interpreting rapid antigen test kits, handling swabs, answering questions from community members). We evaluated perceptions of the most challenging aspects of training (dependent variable) by CHWs and associations between demographics and reported challenges in digital access and literacy among CHWs through univariate regression. We also evaluated associations between self-assessed improvements in digital literacy and demographics among CHWs using multivariable regression. Our study design is summarized in Figure 1.

### Statistical analysis of outcomes and demographics

We assessed the association of indicators of digital literacy within 2 populations: people testing for COVID-19 and among CHWs providing testing. We used multivariable logistic regression for binary outcomes: digital receipt of COVID-19 result and email availability for receipt of COVID-19 result. Time to digital receipt of COVID-19 result was a continuous, quantitative variable that was adjusted for using multivariable quantile regression, due to the presence of outliers. All 3 analyses were adjusted for by multiple demographic variables: age, race, spoken language, sex, CHW affiliation, testing site, and email availability (except for when the outcome was email availability). Given the large percentage of Latinos among the population tested, self-reported race and ethnicity were categorized as Latino, White, and all other races. Given different generations’ experience with technology, age groupings were provided for the multivariable quantile regression using current definitions for generations (Generation Z, Millenials, Gen X, and Boomers). For the analysis of CHWs providing COVID-19 testing, univariate logistic regression was performed for the outcome “Reporting a tech-related issue as most difficult job activity,” given the small number of respondents and multiple variables assessed for association: age, preferred language, age, testing site location, and education level. For reporting improvement of technical skills due to COVID-19 technical training, the outcomes allowed for multiple selections among improvements in laptop use, tablet use, and use of printers. For this reason, multivariable logistic regression was used to control for age, preferred language, testing site location, and education level. We considered a *P*-value of <.05 to be statistically significant. Analyses were conducted in R (R Studio), version 4.1.1.

### Ethics statement

In the COVID-19 testing analyses, all participants ≥18 years of age provided verbal informed consent prior to participation in testing and associated surveys. For children 2-17 years of age, a parent/guardian provided verbal consent for participation. In addition, children 7-12 years of age provided verbal assent and children 13-17 years of age provided verbal informed consent, prior to participation. All CHWs provided written or electronic informed consented before participating in this study. The University of California, San Francisco (UCSF) Committee on Human Research (ie, UCSF’s institutional review board) approved the study protocol, informed consent forms, and study surveys.

## Results

### Community member testing and digital access

Among the 5044 people who tested at Aliados por la Salud sites between March 2021 and March 2022, the median age was 30 years (IQR: 15-44), 53% were women, 91% were Latino, 43% provided an email address for results receipt, and 56% accessed results digitally (by SMS or email). Accessing and viewing results digitally (by SMS or email) vs non-digitally was significantly associated with younger age (Adjusted OR: 0.98 [95% CI, 0.97, 0.99] for each additional year of age), providing an email to access results (OR: 2.18 [95% CI, 1.80, 2.65]), and testing at the rural Planada site (OR: 1.61 [95% CI, 1.30, 1.99]). Providing an email address vs not providing an email address at testing registration was significantly associated with younger age (OR: 0.96 [95% CI, 0.95, 0.97] for each additional year of age), White race (OR: 5.38 [95% CI, 3.25, 8.93] vs non-White), being a monolingual English speaker (OR: 2.38 [95% CI, 1.89, 3.03] vs speaking another language at home), identifying as female (OR: 1.33 [95% CI, 1.10, 1.59]), and testing at the rural Planada site (OR: 3.03 [95% CI, 2.45, 3.76]) (Table 1). Among persons accessing their testing results digitally, the median time from COVID-19 testing to viewing results was 41 min. Increased time from testing to viewing results was significantly associated with older age (quantile regression coefficient for 41-55 years of age: 4.0 [95% CI, 1.15, 6.85], and quantile regression coefficient for > 55 years of age: 10.0 [95% CI, 4.35, 15.65], compared to 18-40 years of age) and testing at the rural Planada site (coefficient: 9.0 [95% CI, 5.5, 12.5]) (Table 2), among persons viewing their results digitally.

**Table 1.** Association between participant demographics and provision of email for accessing result and digital receipt of COVID-19 result (SMS and/or email).

	Overall (*N* = 5044)	Digital receipt of COVID-19 result			Email available for result receipt		
		Yes *N* = 2773	No *N* = 2271	Adjusted OR (95% CI) [*P*-value]	Yes *N* = 2000	No *N* = 3044	Adjusted OR (95% CI) [*P*-value]
Age Median [Min, Max]	30 [2, 96]	29 [2, 90]	32 [2, 96]	**0.98** (0.97, 0.99) for each 1-year increase in age [<.001]^a^	27 [2, 87]	33 [2, 96]	**0.96** (0.95, 0.97) for each 1-year increase in age [<.001]^a^
Race/ethnicity							
Latino	4556 (93.2%)	2476 (92.1%)	2080 (94.5%)	**(Ref)**	1708 (88.8%)	2848 (96.0%)	**(Ref)**
Other	114 (2.3%)	73 (2.7%)	41 (1.9%)	**1.53** (0.83, 2.80) [.170]	76 (4.0%)	38 (1.3%)	**2.93** (1.58, 5.42) [<.001]^a^
White	220 (4.5%)	140 (5.2%)	80 (3.6%)	**1.35** (0.86, 2.12) [.189]	139 (7.2%)	81 (2.7%)	**5.38** (3.25, 8.93) [<.001]^a^
Language other than English spoken at home							
No	620 (21.9%)	364 (22.4%)	256 (21.2%)	**(Ref)**	362 (32.1%)	258 (15.1%)	**(Ref)**
Yes	2215 (78.1%)	1263 (77.6%	952 (78.8%)	**1.19** (0.94, 1.51) [.155]	766 (67.9%)	1449 (84.9%)	**0.42** (0.33, 0.53) [<.001]^a^
Sex							
Female	2577 (51.9%)	1452 (53.1%)	1125 (50.5%)	**(Ref)**	1091 (55.2%)	1486 (49.8%)	**(Ref)**
Male	2384 (48.1%)	1281 (46.9%)	1103 (49.5%)	**0.95** (0.82, 1.11) [.547]	884 (44.8%)	1500 (50.2%)	**0.75** (0.63, 0.91) [.003]^a^
CHW affiliation							
No	2481 (95.4%)	1428 (95.4%)	1053 (95.4%)	**(Ref)**	932 (93.0%)	1549 (96.9%)	**(Ref)**
Yes	120 (4.6%)	51 (4.6%)	69 (4.6%)	**0.78** (0.51, 1.20) [.264]	70 (7.0%)	50 (3.1%)	**1.69** (1.08, 2.63) [.02]^a^
Site							
Canal (peri-urban)	3958 (78.5%)	2066 (74.5%)	1892 (83.3%)	**(Ref)**	1396 (69.8%)	2562 (84.2%)	**(Ref)**
Planada (rural)	1086 (21.5%)	707 (25.5%)	379 (6.7%)	**1.61** (1.30, 1.99) [<.001]^a^	604 (30.2%)	482 (15.8%)	**3.03** (2.45, 3.76) [<.001]^a^
Email available							
No	3044 (60.0%)	1445 (52.1%)	1599 (70.4%)	**(Ref)**			
Yes	2000 (40.0%)	1328 (47.9%)	672 (29.6%)	**2.18** (1.80, 2.65) [<.001]^a^			

The bolded numbers refer to adjusted odds ratios and the (ref) refers to a variable value that provides the reference value for which to compare to other values.

a *P* < .05.

**Table 2.** Association between patient demographics and time to retrieval of results from testing administration (in minutes), among persons testing who accessed their results digitally (quantile regression).

	Overall (*N* = 2733)	Unadjusted minutes from testing to viewing results Median (IQR)	Adjusted coefficient (95% CI) minutes [*P*-value]
Age (in years)			
0-17	757 (27.3%)	40 (32-54)	**−1.0** (−6.03, 4.03) [.697]
18-40	1212 (43.7%)	39 (31-55)	**(ref)**
41-55	581 (21.0%)	42 (33-65)	**4.0** (1.15, 6.85) [.006]^a^
>55	223 (8.04%)	49 (34-81)	**10.0** (4.35, 15.65) [.001]^a^
Race/ethnicity			
Latino	2476 (92.1%)	41 (32-60)	**(ref)**
Other	73 (2.71%)	38 (29-63)	**−3.0** (−12.0, 6.0) [.516]
White	140 (5.21%)	40 (32-51)	**−3.0** (−7.5, 1.5) [.187]
Language other than English spoken at home			
No	364 (22.4%)	43 (33-67)	**(ref)**
Yes	1263 (77.6%)	41 (32-65)	**−2.0** (−6.6, 2.6) [.396]
Sex			
Female	1452 (53.1%)	41 (32-62)	**(ref)**
Male	1281 (46.9%)	39 (32-57)	**−1.0** (−3.2, 1.2) [.368]
CHW affiliation			
No	1428 (95.4%)	41 (32-63)	**(ref)**
Yes	69 (4.61%)	54 (27-110)	**15.0** (−3.0, 33.0) [.102]
Site			
Canal (peri-urban)	2066 (74.5%)	39 (31-55)	**(ref)**
Planada (rural)	707 (25.5%)	46 (35-70)	**9.0** (5.5, 12.5) [<.001]^a^
Email available			
No	1445 (52.1%)	40 (31-58)	**1.0** (−3.3, 1.3) [.399]
Yes	1328 (47.9%)	41 (32-61)	**(ref)**

The bolded numbers refer to adjusted odds ratios and the (ref) refers to a variable value that provides the reference value for which to compare to other values.

a *P* < .05.

### CHWs and digital literacy

Forty-two of 52 CHWs (81%) participating in COVID-19 testing activities completed the technology skills assessment survey. Among the 42 CHWs, the median age was 44 (IQR: 30-52), 90% were women, and 56% completed high school or more. Sixty-eight percent of the CHWs reported technology-related challenges as the most difficult aspect of providing COVID-19 testing services. CHW reporting that digital technology was the most challenging aspect of providing testing was associated with older age (OR: 1.06 [95% CI, 1.01, 1.12] *for each 1-year increase in age*) and working in the rural community of Planada compared to Canal (peri-urban) (OR: 5.91 [95% CI, 1.41, 24.73]). We did not observe a significant association between preferred language (Table 3) and technology-related challenges. Following training in use of digital technology for COVID-19 testing service delivery, 71% of CHWs reported improvement in using laptops for data entry, and 66% reported improvement in tablet use. When asked about improvement of technical skills (Table 4), CHWs from the rural study site (Planada) had decreased odds of reporting improving skills in laptop use for data entry (OR: 0.04 [95% CI, 0.01, 0.56]) and use of printers (OR: 0.03 [95% CI, 0.002, 0.35]) when compared to CHWs from the peri-urban site (Canal). Self-reported skill improvement with use of tablets was associated with higher age (OR: 1.1 [95% CI, 1.0, 1.2]).

**Table 3.** Proportion of community health workers reporting a digital technology-related challenge vs non-digital technology-related challenge as most difficult job activity.

Demographics	Non-tech related *N* = 13 (row%)	Tech related *N* = 29 (row%)	Total CHWs *N* = 42 (col%)	Unadjusted OR (95% CI)	*P*-value
Age Median [Min, Max]	38 [20, 51]	50 [19, 70]	**44 [19, 70]**	*1.06 (1.01, 1.12)* for each 1-year increase in age	.018^a^
Preferred language					
English	**5** (39%)	**8** (61%)	**13** (31%)	*(ref)*	(ref)
Spanish	**8** (28%)	**21** (72%)	**29** (69%)	*1.64 (0.41, 6.54)*	.49
Location					
Canal (peri-urban)	**9** (53%)	**8** (47%)	**17** (41%)	*(ref)*	(ref)
Planada (rural)	**4** (16%)	**21** (84%)	**25** (59%)	*5.91 (1.41, 24.73)*	.011^a^
Education level^a^					
<12 grade	**3** (30%)	**7** (70%)	**10** (25%)	*(ref)*	(ref)
High school	**6** (33%)	**13** (67%)	**19** (46%)	*0.93 (0.18, 4.9)*	*.93*
Bachelors/higher	**4** (36%)	**8** (64%)	**12** (29%)	*0.86 (0.14, 5.23)*	*.867*

The bold number under Total CHW refers to the age median and min, max values. The other bolded numbers refer to total Ns for each category. The italics refers to Unadjusted OR (95% CI).

a *P* < .05.

**Table 4.** Proportion of community health workers reporting improvement in technical skills due to COVID-19 testing training.

	Improvement in laptop for data entry skills			Improvement in tablet use			Improvement in label/paper printer use		
	Yes *N* = 12	No *N* = 30	OR (95% CI) [*P*-value]	Yes *N* = 28	No *N* = 14	OR (95% CI) [*P*-value]	Yes *N* = 18	No *N* = 24	OR (95% CI) [*P*-value]
Age (median)	41.5	50	**1.03** (0.96, 1.11) [.433] for each 1-year increase in age	49	28	**1.1** (1.0, 1.2) [.043]^a^ for each 1-year increase in age	38.5	50	**0.96** (0.89, 1.04) [.35] for each 1-year increase in age
Education level completed									
<12 grade	8 (80%)	2 (20%)	(ref)	9 (90%)	1 (10%)	(ref)	3 (30%)	7 (70%)	(ref)
High school	15 (79%)	4 (21%)	**1.1** (0.12, 10.1) [.93]	11 (58%)	8 (42%)	**0.20** (0.02, 2.5) [.21]	7 (37%)	12 (63%)	**3.5** (0.34, 35.5) [.29]
Bachelors/higher	6 (50%)	6 (50%)	**0.1** (0.01, 1.75) [.115]	7 (58%)	5 (42%)	**0.38** (0.02, 7.5) [.52]	7 (58%)	5 (42%)	**30.5** (1.03, 907.8) [.05]^a^
Preferred language									
English	10 (77%)	3 (23%)	(ref)	5 (38%)	8 (62%)	(ref)	8 (62%)	5 (38%)	(ref)
Spanish	20 (69%)	9 (31%)	**0.1** (0.01, 1.4) [.09]**	23 (79%)	6 (21%)	**1.81** (0.2, 20.6) [.633]	10 (34%)	19 (66%)	**0.8** (0.06, 10.9) [.811]
Location									
Canal (peri-urban)	16 (94%)	1 (6%)	(ref)	13 (76%)	4 (24%)	(ref)	12 (71%)	5 (29%)	(ref)
Planada (rural)	14 (56%)	11 (44%)	**0.04** (0.01, 0.56) [.02]^a^	15 (60%)	10 (40%)	**0.16** (0.02, 1.24) [.08]	6 (24%)	19 (76%)	**0.03** (0.002, 0.35) [.005]^a^

The bold number refers to ORs.

a *P* < .05. ** *P*<.10.

## Discussion

In this study, characterizing the use of digital methods to access COVID-19 test results among residents of low-income, majority-Latino communities in Northern California, we found that nearly half (44%) of community members did not access results digitally (by SMS or email). We also found that provision of an email address at registration was associated with digitally accessing results. In addition, we examined the impact of digital literacy training on self-reported improvements in technology use among CHWs offering COVID-19 testing services through digital platforms. We found that CHWs from rural communities had relatively higher odds of reporting technology-related challenges, and lower odds of reporting improvement in technology use after training, compared to CHWs from semi-urban communities. While integrating CHWs into COVID-19 testing interventions is essential to advancing equity,^21^ they may also require intensive training and ongoing support.

Community member provision of an email address for accessing COVID-19 test results may be an important indicator of digital access and literacy among underserved populations, such as the majority-Latino communities in this study. Though we did not find examples of email address provision as a marker of digital access in other studies of digital literacy and public health interventions, United Nations Educational, Scientific and Cultural Organization (UNESCO) has identified availability and use of an email address as an important indicator of digital literacy competency.^22^ Notably, as a single indicator, email address provision was significantly associated with several demographic variables that have previously been associated with decreased digital access and literacy: race, age, language, and rural location.^8^ The provision of an email address was also associated with increased odds of accessing COVID-19 results digitally. Email address provision may also serve as a simple metric for identifying members of the testing population that require additional assistance and outreach when offering testing via digital platforms, to ensure equitable and timely access to results.

Rural residence has been an important predictor of digital literacy in prior studies,^23^^,^^24^ with differential effects based on race,^25^ given its association with structural factors such as broadband internet availability. The digital divide has long been present in rural communities, and has persisted despite rural-focused interventions,^23^^,^^24^ resulting in limited participation in quick digitization of health services, work, and school during the COVID-19 pandemic.^26^ Notably, we found that rural residence was a significant factor across all of the outcomes we analyzed, but not always in the anticipated or same direction. While those tested for COVID-19 in rural settings had significantly increased time to results viewing compared to peri-urban settings, they also had increased odds of providing emails for result notification and accessing results electronically compared to persons testing in peri-urban settings. These unexpected findings may have been due to digital literacy workshops organized and funded by the county government that took place during the study period, independently of our intervention in the rural community. The small absolute difference in time in accessing results (9 min in unadjusted analysis) is unlikely to be of public health significance in this case, but may have public health implications in other contexts. Despite some differences in associations between rural residence and digital literacy by study outcome, overall we found that rural residence remained associated with increased time to results receipt (among those accessing results digitally) among persons testing for COVID-19, and greater difficulty in use of digital tools during testing implementation among CHWs. These findings suggest that rural areas continue to need greater attention, but may be favorably targeted by interventions to bridge the digital divide and ensure equitable access to digital health platforms.

CHWs have long been instrumental in lower- and middle-income countries (LMICs) with increasing relevance in the United States for providing connected community support to increase positive health outcomes in identifying and managing both communicable and chronic diseases.^27^^,^^28^ Reviews of digital technology use by CHWs for the pandemic response in LMICs have highlighted the need to provide training to CHWs in digital tool use and digital literacy to take advantage of the benefits of digital technology and health.^29–31^ Despite this need, there has been limited literature on evaluating CHW self-reported digital literacy in the United States, and capacity building in response to training.^14^ To support equitable access to COVID-19 testing services,^21^ we trained CHWs on delivery of COVID-19 testing using our digital platforms and tools. Rural and older CHWs were more likely to report technology-related challenges in providing COVID-19 testing, suggesting a need for more intensive training in these demographic groups when rolling out digital health platforms. Of note, in our peri-urban community (the Canal, San Rafael), digital interventions during the study period promoted free and low-cost internet access for community members, gifted laptops for schooling, and offered services to increase digital literacy. This may have led to CHWs from the peri-urban setting reporting increased technical skills when compared to the rural site, although survey questions asked specifically about skill improvement related to COVID-19 testing training. Older CHWs were more likely to report greater improvement in digital literacy after training, possibly suggesting that they may have started with a greater deficit in digital skills. These findings highlight the ongoing importance of evaluating digital training needs, as well as building on digital literacy interventions already occurring in these communities.

Our study has limitations. First, in some instances, non-provision of an email address may have been for reasons other than lacking an email address (ie, privacy concerns). However, given the associations we observed between provision of email and demographics commonly associated with the digital divide, as well as with its association with accessing digital results, we believe email provision may serve as an important first-pass indicator of testers that require more assistance. Some of the testing population may have been hesitant to share email addresses, given possible undocumented immigration status and fear of registration. However, this was thought to be ameliorated through CHW assistance in registration and testing. Email addresses may also have been shared between family members in some cases, and indeed, we identified instances where the same email address was provided by more than 1 member of a family, which could have resulted in overestimation of the digital literacy indicator of residents providing an email at time of testing. However, instances of shared email, where 1 family member is responsible for receiving and sharing digital COVID-19 results with others in a family or household, are still likely to represent a form of digital health access. Furthermore, among young (<11-year-old) testing populations, a parent or guardian likely viewed the COVID-19 test results, which may have affected our analyses of associations between demographics and results receipt. However, in sensitivity analyses that excluded participants <11 years of age, we found no appreciable differences in our results (data not shown). Second, in assessing the impact of digital training among CHWs, we relied on self-report rather than objective measures of proficiency on the digital platform used for COVID-19 testing. However, we believe that these cross-sectional surveys remain useful to rapidly assess needs and effects of digital training. Finally, we evaluated CHWs in a cross-sectional survey, rather than assessing changes in perceived digital technology use over time. As such, we may have evaluated a slightly different cohort of CHWs than those present at the beginning of the project, resulting in variable amounts of digital training among CHWs surveyed. However, as there was routine reinforcement of trainings and subsequent re-trainings, we believe that slight changes in CHW participation over the course of the study are unlikely to have had a meaningful impact on the CHW survey findings.

Our findings provide additional insights into efforts to ensure more equitable community-based testing for COVID-19 via digital platforms in the context of the digital divide. As public health interventions for current and future pandemics become increasingly digitized, it will be important to support traditionally underserved populations that may need assistance to realize the full impact of digital health interventions, and to understand how best to support CHWs working to address public health inequities in an era of digital health transformation.

## Data Availability

The data underlying this article will be made available, with access details provided once the data are complete.
